# Improving hospital-based point-of-care ultrasound cleaning practices using targeted interventions: a pre–post study

**DOI:** 10.1186/s13089-021-00244-4

**Published:** 2021-10-18

**Authors:** Daniel Van Kalsbeek, Karl Enroth, Elizabeth Lyden, Mark E. Rupp, Christopher J. Smith

**Affiliations:** 1 Department of Internal Medicine, 982055 Nebraska Medical Center, Omaha, NE 68198-2055 USA; 2 Department of Biostatistics, 984375 Nebraska Medical Center, Omaha, NE 68198-4375 USA; 3 Department of Internal Medicine, Division of Infectious Diseases, 985400 Nebraska Medical Center, Omaha, NE 68198-5400 USA; 4 Department of Internal Medicine, Division of Hospital Medicine, 986430 Nebraska Medical Center, Omaha, NE 68198-6430 USA

**Keywords:** Point-of-care ultrasound (POCUS), Infection control, Cleaning, Disinfection, Quality improvement

## Abstract

**Background:**

Point-of-care ultrasound (POCUS) devices are becoming more widely used in healthcare and have the potential to act as fomites. The objective of this project was to study the thoroughness of cleaning of POCUS machines before and after a quality improvement initiative. We designed a mixed-methods, pre/post study which took place over the course of one year at a university-affiliated health center. Cleaning rates of four ultrasound machines used by hospital medicine and critical care medicine services were evaluated using fluorescent marking. Interventions targeted physicians’ knowledge of best practices and improved access to cleaning supplies. Pre- and post-intervention cleaning rates were compared using a generalized linear model. The impact of the corona virus disease of 2019 (COVID-19) pandemic on baseline cleaning rates was also evaluated. Physicians’ attitudes and knowledge of cleaning practices were evaluated via unpaired pre/post surveys.

**Results:**

There was significant improvement in thoroughness of cleaning following intervention (pre 0.62, SE 0.05; post 0.89, SE 0.07), *p* < 0.0001). There was no difference in baseline cleaning rates before (0.63, SE 0.09) and after (0.61, SE 0.1) the onset of the COVID-19 pandemic (*p* = 0.78). Post-intervention surveying found improved understanding of guideline-based cleaning practice, better performance on knowledge-based questions, and fewer reported barriers to machine cleaning.

**Conclusion:**

Thoroughness of cleaning of POCUS machines can be improved with practical interventions that target knowledge and access to cleaning supplies.

**Supplementary Information:**

The online version contains supplementary material available at 10.1186/s13089-021-00244-4.

## Background

Over the past three decades, the use of point-of-care ultrasound (POCUS) has been increasingly integrated into routine clinical practice, including critical care and hospital medicine [1–3]. Recently the Alliance for Academic Internal Medicine has advocated for POCUS training across all levels of medical education [3]. Advances in technology have allowed ultrasound machines to become more portable and user-friendly, making it possible to perform exams on multiple patients in a relatively short time frame [2, 4]. Clinically relevant infectious organisms have been cultured from ultrasound probes and gels, even when they are not visibly soiled, including methicillin-resistant *Staphylococcus aureus*, *Pseudomonas aeruginosa* and vancomycin-resistant enterococci [5–9]. Additionally relevant to the present time, it has been discovered that SARS-CoV-2 may remain viable on plastic surfaces for up to 72 h [10]. Thus, POCUS has the potential to act as a fomite for pathogens which are known to prolong hospital stays, escalate healthcare costs, and increase mortality [11].

Cleaning and disinfection of ultrasound equipment has been shown to significantly reduce microbiological burden [12, 13]. Multiple professional groups have published guidelines for cleaning and disinfecting POCUS machines, and, though difficult to prove due to study design, adherence to these guidelines should reduce the risk of transmitting clinically relevant pathogens [14–21]. Unfortunately, surveys suggest suboptimal cleaning practice and a perceived lack of guidance among ultrasound users [22–24]. Hospital-based studies have shown that environmental cleaning performance can be improved using education and feed-back, leading to reduced likelihood of culturing clinically relevant organisms [20, 21, 25–27]. Similar studies pertaining to point-of-care ultrasound are lacking. To address this, we designed a project with the goal of identifying barriers to guideline-based cleaning of POCUS devices and improving cleaning practices using targeted interventions.

## Methods

### Design and setting

A mixed-methods, pre/post-study of POCUS machine cleaning practices was conducted over a 1-year period at a 718-bed academic health center. This project was considered quality improvement by the local institutional review board and therefore did not require approval as human subject research. Two Philips SPARQ (Andover, MA) and 2 GE Healthcare Venue (Chicago, IL) machines were included in the study. SPARQ machines were primarily used by hospital medicine (HM) faculty and internal medicine (IM) residents. One SPARQ machine was stored on an inpatient medical-surgery unit and the other was stored in a HM work room. Venue machines were used primarily by IM residents, pulmonary/critical care medicine (PCCM) fellows, and PCCM faculty, but they were also accessible to surgical and anesthesia critical care providers. One Venue was stored in a neuro-critical care unit and the other was stored in a medical critical care unit. All machines were outfitted with linear, convex, and phased array transducers.

The IM residency program has approximately 95 house officers, including categorical, preliminary, primary care, and medicine-pediatric residents. The residency program has a longitudinal POCUS curriculum, including workshops, lectures, and a dedicated POCUS rotation. There are 13 PCCM fellows, who also have a dedicated POCUS curriculum and rotation. POCUS training and clinical use is variable for HM and PCCM faculty, with some high-frequency users and some non-users.

### Intervention

Interventions were guided by a targeted needs assessment. To better educate physicians, we created a website that outlined best practices for POCUS cleaning and disinfection [28]. The website included a 3-min instructional video, a summary of cleaning steps, and links to best-practice guidelines from our institution and relevant professional societies. The website was distributed via email to all resident, fellow, and faculty physicians targeted by our survey. The instructional video was also integrated into training sessions for the IM residents and PCCM fellows. Placards were placed on POCUS machines which included a summary of cleaning steps and a QR code to access the website for just-in-time training.

The educational intervention promoted a 2-step cleaning and disinfection process based on American Institute of Ultrasound in Medicine guidelines [14]. First, users were instructed to remove all visible soiling with a disinfecting wipe or soapy washcloth. Then, using a fresh disinfecting wipe, all high-use areas (touch screen, keyboard, handles, probes, and cords) were wiped thoroughly. PDI Sani-Cloth^®^ AF-3 disinfecting wipes were used throughout the study. This product is a quaternary ammonium-containing, low-level disinfectant, which is appropriate for disinfecting medical equipment that contacts intact skin [29].

**Fig. 1:**
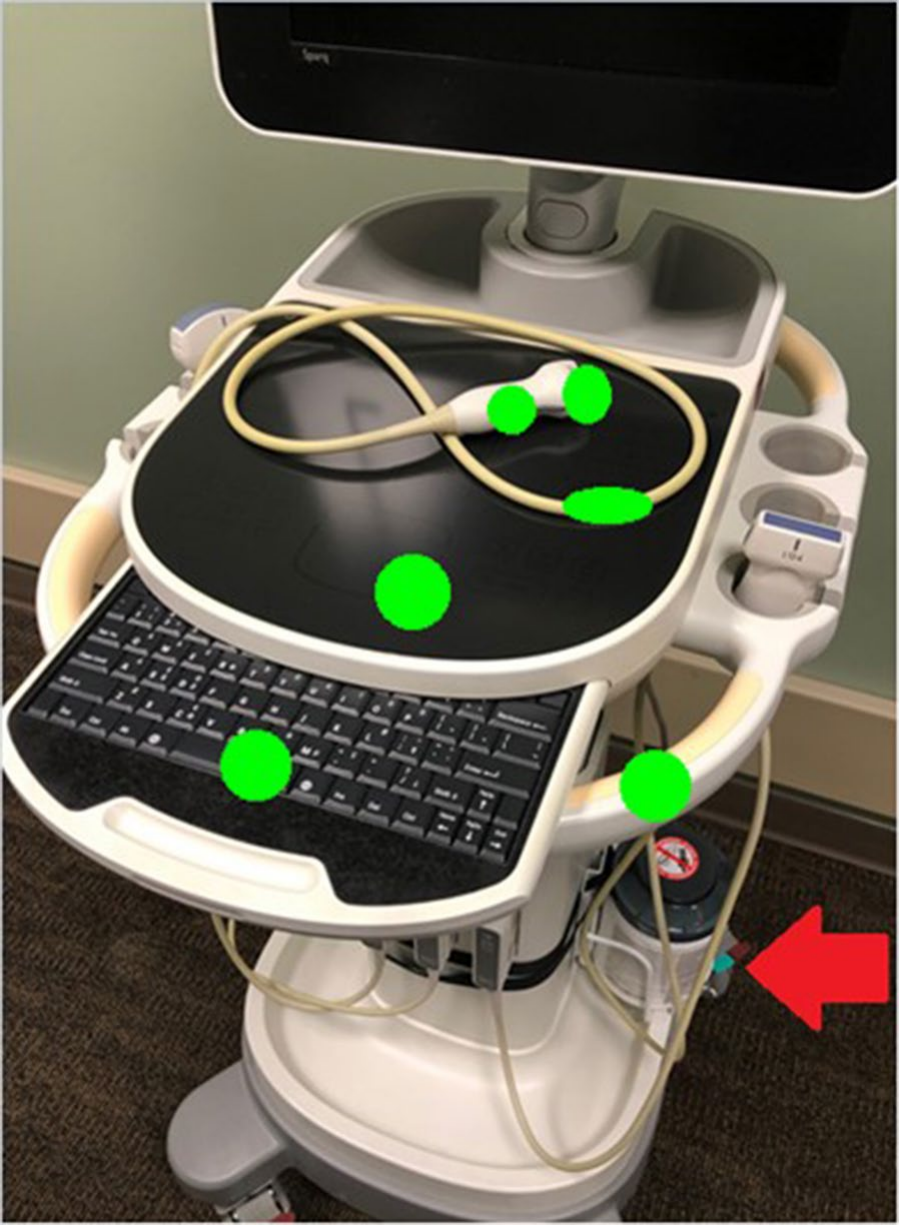
Locations of fluorescent marks. A Phillips SPARQ model ultrasound machine is pictured to demonstrate marking locations. Green circles represent the areas marked with fluorescent gel during the study (size exaggerated for clarity). For simplicity, only one probe and cord is displayed, but all probes and cords were marked in this fashion during the study. Also pictured is the disinfecting wipe caddie that was added to these models to improve access to cleaning supplies (red arrow)

We improved access to cleaning materials by ensuring that each machine always had a dedicated container of disinfecting wipes. Machines with inadequate space for wipes were equipped with commercially available canister caddies (Fig. 1). Wipes were restocked by researchers twice weekly, as needed, during study periods. Providers were also encouraged to replace wipes when necessary.

### Data collection

**Fig. 2:**
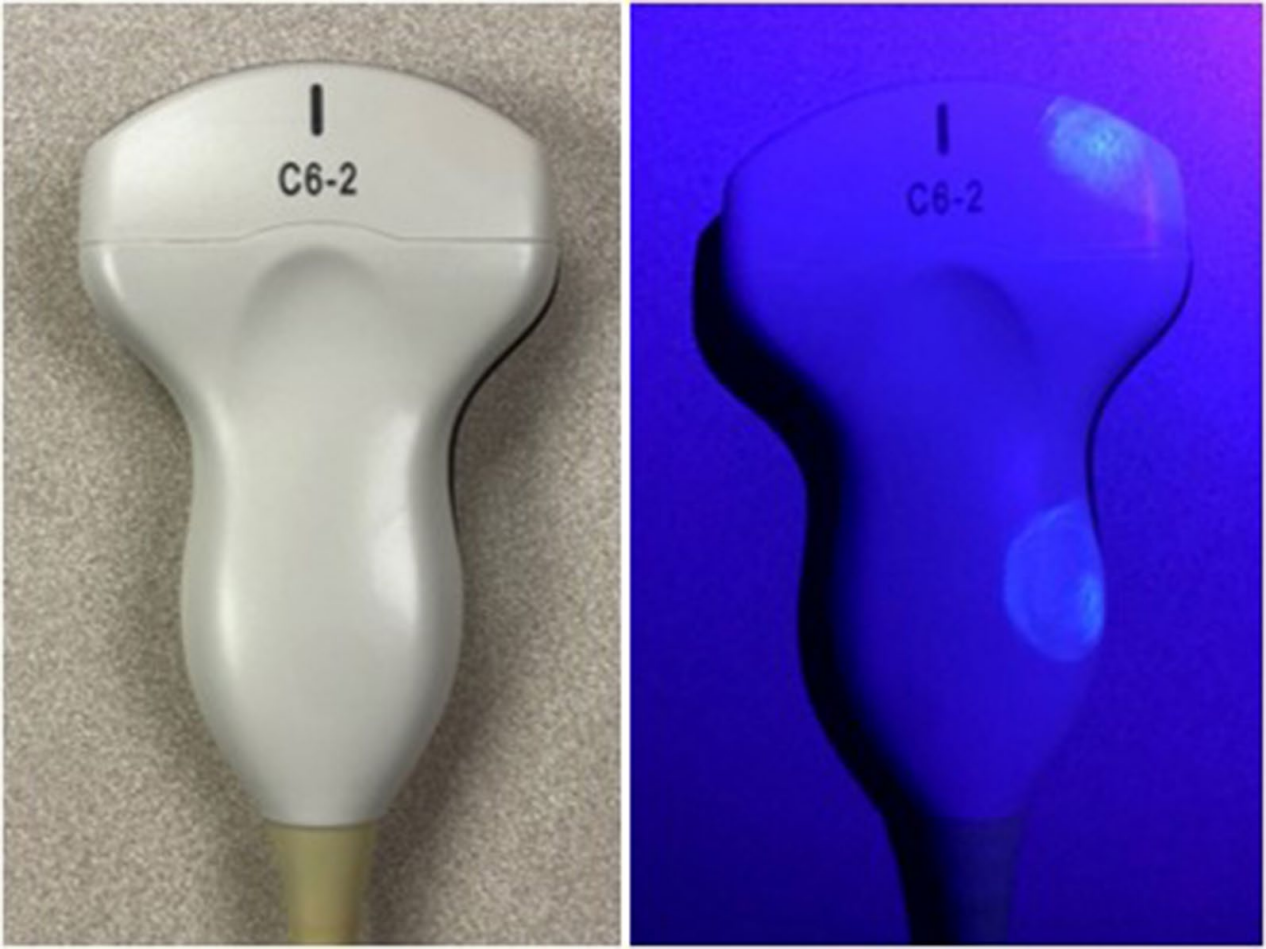
Fluorescent gel marks. Pictured is a curvilinear probe used to exemplify the size and location of fluorescent marks under ambient room light (left) and UV light with room lights turned off (right)

A fluorescent marking gel (DAZO, ECOLAB) was used to monitor POCUS machine cleaning practices. The use of these gels is a well-established method for monitoring environmental cleaning practices in infection control research [25–27, 30, 31]. Circular marks roughly 2.54 cm in diameter were placed on 12 high-touch areas on each machine including probes, cords, and user-interface surfaces (Figs. 1 and 2). The marking sites were determined based on best practice recommendations [14, 15] and guidance from our infection control department. The size of gel markings were informed by pilot testing which revealed this pattern allowed the most consistent application of gel. Gel markings were evaluated twice per week during the first two weeks of each month during the study period by members of the research team (DV and KE). The absence of fluorescence under UV light was classified as cleaned. The presence of any visible fluorescence was classified as dirty. This was done to minimize subjectivity when interpreting partially cleaned marks and because residual fluorescence was felt to indicate poor cleaning technique. The presence of gross debris, defined as the presence of soiling that was visible to the naked eye, was also documented. The machines were thoroughly cleaned per institutional guidelines and evaluated with UV light to ensure no remaining fluorescence remained prior to re-marking. Prior to the start of the project, researchers met to ensure machine marking and data collection procedures were consistent between team members. Machine use was tracked via a dedicated sign-out sheet for each machine.

The pre-intervention data collection occurred from November 2019 to January 2020. Before implementation of the planned intervention, the COVID-19 pandemic spread rapidly across the United States. The heightened awareness around infection control during this time had the potential to confound post-intervention data. To address this possibility, the research team collected an additional 2 months of baseline data from May to June 2020. During this time only three of the four POCUS machines were available for data collection. Interventions were implemented in July 2020. Post-intervention data were collected from August to October 2020.

### Survey

A 16-question survey was created to assess participants’ demographics, POCUS experiences, and attitudes and knowledge towards machine cleaning (Additional file 1). The survey was created through an iterative process with input from a survey design expert within the institution. It was pilot tested by three members of a POCUS interest group (one faculty, two residents) to ensure clarity, ease of use, and face validity. The survey was distributed to all IM residents; fellows from PCCM & Rheumatology; and faculty from HM, PCCM, and Rheumatology with POCUS training. Rheumatology physicians were included as they routinely use POCUS in clinical practice at our institution. A link to the anonymous, online survey was distributed via institutional email in April 2020 (pre-intervention) and October 2020 (post-intervention).

### Analysis

The primary outcome was the composite machine cleaning rates in the pre-intervention phase compared to the post-intervention phase. Secondary outcomes included comparison of cleaning rates based on primary machine ownership (HM vs PCCM) and marking site groups (transducers vs. cords vs. touch surfaces). Cleaning rates were analyzed using a generalized linear mixed model (GLMM) which accounted for the correlation of cleaning rates for each machine. Model-adjusted mean percentages and standard errors of cleaned components by phase were used to summarize the data. To account for occasions when a machine was not used between sampling periods, data were excluded when there was no documentation of use on the machine’s sign-out sheet and there was no evidence of use (i.e., all gel marks remained intact and no gross debris was present). This was done to avoid falsely under-estimating cleaning rates. Unpaired pre- and post-intervention survey results were compared using descriptive statistics. All analyses were done using SAS version 9.4, and a *p*-value of less than 0.05 was considered statistically significant.

## Results

There was no difference in pre-intervention cleaning rates before (0.63, SE 0.09) and after (0.61, SE 0.1) the COVID-19 outbreak (*p* = 0.78). These data were, therefore, combined to form an aggregate baseline cleaning rate that was used in the final analysis. Pre- and post-intervention cleaning rates are displayed in Table 1. A total of 768 surfaces were evaluated in the pre-intervention phase (504 pre-COVID, 264 post-COVID) and 504 surfaces in the post-intervention phase. There was a significant improvement in the thoroughness of cleaning following intervention (pre 0.62, SE 0.05; post 0.89, SE 0.07; *p* < 0.0001), representing a 44% relative improvement. In a sub-group analysis, the HM-based machines (pre 0.50, SE 0.08; post 0.91, SE 0.14; *p* < 0.0001) demonstrated greater improvement in the thoroughness of cleaning than the CCM-based machines (pre 0.74, SE 0.04; post 0.87, SE 0.04; *p* = 0.075). All surface category sub-groups showed significant improvement in cleaning rates (*p* ≤ 0.01 for all groups), with probe cords showing the largest improvement (Table 1). During the pre-intervention phase there were 12 instances where gross debris was found on the machines, while there was a single instance in the post-intervention phase. All instances of gross debris were found on probe lenses and/or handles. While not specifically analyzed, the gross debris visually appeared to be an accumulation of dried gel, often associated with a designed crevice such as the probe orientation marker. There was a single instance where blood appeared to be intermixed in the gross debris.

**Table 1:** Model-adjusted ultrasound machine cleaning rates before and after quality improvement interventions

	Before (SE)	After (SE)	*p*-value
All machines combined	0.62 (0.05)	0.89 (0.07)	*p* < 0.0001
Primary machine usage sub-groups			
Hospital medicine machines	0.50 (0.08)	0.91 (0.14)	*p* < 0.0001
Critical care machines	0.74 (0.04)	0.87 (0.05)	*p* = 0.075
Surface category sub-groups			
Probe lens and handles	0.74 (0.06)	0.96 (0.07)	*p* < 0.0001
Probe cords	0.40 (0.06)	0.78 (0.10)	*p* < 0.0001
High-touch surfaces	0.63 (0.06)	0.88 (0.08)	*p* = 0.011

Table 2 displays the pre- and post-intervention survey results. The response rates for the pre- and post-intervention surveys were 54% (74/137) and 42% (58/139), respectively. The majority of respondents were IM residents (66%). Roughly half of respondents used POCUS weekly or more. Lack of knowledge, limited access to cleaning supplies, and time constraint were identified as the most common barriers to guidelines-based POCUS cleaning and disinfection. Following the intervention, self-reported familiarity with machine cleaning best practices improved from 22 to 74%. The proportion of participants correctly answering knowledge-based questions related to disinfectant use (pre—59%, post—74%) and cleaning processes (pre—76%, post—93%) also improved. Participants reported fewer barriers related to cleaning supply access, time constraints, and knowledge deficits following the intervention.

**Table 2:** Ultrasound machine cleaning survey results before and after quality improvement interventions

Survey topic	Before (*n* = 74)	After (*n* = 58)
Female sex, *n* (%)	35 (47%)	23 (40%)
Level of training, *n* (%)		
Internal medicine resident	51 (69%)	36 (62%)
Fellow	6 (8%)	7 (12%)
Faculty	17 (23%)	15 (26%)
Faculty/fellow area of practice, *n* (%)		
Hospital medicine/general internal medicine	9 (39%)	9 (41%)
Pulmonary/critical care	8 (35%)	4 (18%)
Rheumatology	6 (26%)	9 (41%)
Frequency of POCUS use, *n* (%)		
Weekly or more	34 (46%)	25 (43%)
Less than weekly	40 (54%)	33 (57%)
Respondents reporting familiarity with best practice guidelines for POCUS cleaning, *n* (%)	16 (22%)	43 (74%)
Respondents reporting they know where to find institutional guidelines for cleaning, *n* (%)	3 (4%)	33 (57%)
My cleaning practices follow best practice guidelines, *n* (%)		
Agree or strongly agree	12 (16%)	34 (76%)
Disagree or strongly disagree	4 (5%)	0 (0%)
I don’t know	58 (78%)	14 (24%)
Correct response to objective knowledge questions, *n* (%)		
Appropriate use of disinfectants	44 (59%)	43 (74%)
Recommended steps of cleaning and disinfecting process	56 (76%)	54 (93%)
Barriers to cleaning, Median Likert (IQR)^a^		
Access to cleaning supplies	3 (2–3)	2 (1–3)
Time constraint	3 (2–3)	2 (1–3)
Lack of knowledge	3 (3–4)	2 (1–3)

^a^5-point Likert scale: 1 = never, 2 = rarely, 3 = sometimes, 4 = often, 5 = always

## Discussion

We were able to significantly improve the thoroughness of cleaning of POCUS machines by using targeted interventions. We identified lack of knowledge, inadequate access to cleaning supplies, and time constraint as primary barriers to guideline-based cleaning and disinfection. These findings support the concern raised by a global survey of ultrasound users which suggested that real-world cleaning practices are unsatisfactory and that users are unfamiliar with guidelines [22, 23]. Given the morbidity, mortality, and cost associated with nosocomial infection, preventative efforts should be pursued whenever feasible [32, 33]. The interventions we described are practical and could be readily implemented at other institutions. A review of guideline-based cleaning and disinfection practice should become a standard component of POCUS training. The effort and expense of such interventions is likely justified if they can prevent even a small number hospital acquired infection [32].

The spread of the COVID-19 pandemic during our pre-intervention phase had the potential to confound our results, for example, through heightened provider awareness of disinfecting practices or system-level interventions. However, we did not find any difference in cleaning rates following the first spike of COVID-19-related admission in our region of the country [34]. This finding is a powerful example of how difficult it can be to change clinical practices. We believe our project was successful because it identified specific physician-reported barriers allowing for tailored intervention [35].

When sub-grouped by area of use, the thoroughness of cleaning for machines used primarily for critical care approached, but did not meet, significance. These machines are shared with specialties that were not targeted by our interventions, providing a possible explanation for this finding. Of the surface category sub-groups, probe cords had the lowest baseline cleaning rate and the greatest relative improvement. While probe cords may not be thought of as a high-contact surface, they are frequently handled during exams or draped across the patient, and attention must be given to this area during cleaning. Though statistical analysis was not performed due to the unpaired nature of our survey, there was an improvement in all response categories following intervention, further supporting the efficacy of these actions.

Although our intervention focused on POCUS users, we would like to highlight an opportunity for manufacturers to improve device design to promote easier cleaning and better infection control. Specifically, there should be efforts to reduce the number of difficult-to-clean crevices, such as we encountered around probe orientation markers. These are in the highest-contact region and we consistently found them to be poorly cleaned. Additional areas of concern include the keyboard and control knobs/wheels.

There were limitations to our study. This was a single-center study, which may limit generalizability. The pre/post-intervention study design makes it possible that confounding variables influenced our findings. Our design allowed measurement of cleaning but not disinfection, although prior infection control studies demonstrated that improvement in thoroughness of cleaning correlated closely with microbiological assessment [20, 21, 26]. Additionally, thoroughness of cleaning was assessed twice per week, making it possible that machines were used multiple times between assessments. This could have falsely increased our cleaning rates, though should not affect the relative change. Despite these potential limitations, we feel our findings provide meaningful and useful data.

Based on the results of this study, guideline-based cleaning practices and education have been integrated into our POCUS curriculum. This includes ongoing use of the online resources created for the project and improved access to dedicated disinfecting wipes on POCUS machines. In the future, we would like to work with our infection control division to update institutional cleaning and disinfection policies so that they are more easily interpretable with regards to POCUS. An internal sustainability study would add important information, and we would be interested in the findings of a similar project carried out at other sites. Finally, demonstrating a correlation between improvement in point-of-care ultrasound cleaning and decreased patient acquisition of microbes or healthcare-associated infection is desirable.

## Conclusion

We identified barriers to guideline-based cleaning and disinfection of point-of-care ultrasound machines: a lack of knowledge, time constraint, and inadequate access to cleaning supplies. By targeting these domains with practical interventions, we were able to significantly improve the thoroughness of cleaning of four point-of-care ultrasound machines used by internal medicine and critical care medicine providers.

## Supplementary Information

**Additional file 1.** Point-of-care ultrasound cleaning survey.

## Data Availability

The datasets used and/or analyzed during the current study are available from the corresponding author on reasonable request.

## References

[1] Beaulieu Y, Marik PE (2005). Bedside ultrasonography in the ICU: part 1. Chest.

[2] Campbell SJ, Bechara R, Islam S (2018). Point-of-care ultrasound in the intensive care unit. Clin Chest Med.

[3] Soni NJ, Schnobrich D, Mathews BK (2019). Point-of-care ultrasound for hospitalists: a position statement of the society of hospital medicine. J Hosp Med.

[4] Bhagra A, Tierney DM, Sekiguchi H, Soni NJ (2016). Point-of-care ultrasonography for primary care physicians and general internists. Mayo Clin Proc.

[5] Frazee BW, Fahimi J, Lambert L, Nagdev A (2011). Emergency department ultrasonographic probe contamination and experimental model of probe disinfection. Ann Emerg Med.

[6] Shokoohi H, Armstrong P, Tansek R (2015). Emergency department ultrasound probe infection control: challenges and solutions. Open Access Emerg Med.

[7] Marigliano A, D'Errico MM, Pellegrini I, Savini S, Prospero E, Barbadoro P (2010). Ultrasound echocardiographic gel contamination by Burkholderia cepacia in an Italian hospital. J Hosp Infect.

[8] Westerway SC, Basseal JM, Brockway A, Hyett JA, Carter DA (2017). Potential infection control risks associated with ultrasound equipment—a bacterial perspective. Ultrasound Med Biol.

[9] Lee AS, White E, Monahan LG, Jensen SO, Chan R, van Hal SJ (2018). Defining the role of the environment in the emergence and persistence of vanA vancomycin-resistant enterococcus (VRE) in an intensive care unit: a molecular epidemiological study. Infect Control Hosp Epidemiol.

[10] van Doremalen N, Bushmaker T, Morris D (2020). Aerosol and surface stability of SARS-CoV-2 as compared with SARS-CoV-1. N Engl J Med.

[11] Cosgrove SE, Qi Y, Kaye KS, Harbarth S, Karchmer AW, Carmeli Y (2005). The impact of methicillin resistance in Staphylococcus aureus bacteremia on patient outcomes: mortality, length of stay, and hospital charges. Infect Control Hosp Epidemiol.

[12] Mullins K, Burnham K, Henricson EK, Cohen S, Fair J, Ray JW (2020). Identification and analysis of bacterial contamination of ultrasound transducers and multiuse ultrasound transmission gel bottle tips before and after the aseptic cleansing technique. J Ultrasound Med.

[13] Mullaney PJ, Munthali P, Vlachou P, Jenkins D, Rathod A, Entwisle J (2007). How clean is your probe? Microbiological assessment of ultrasound transducers in routine clinical use, and cost-effective ways to reduce contamination. Clin Radiol.

[14] AIUM official statement: guidelines for cleaning and preparing external- and internal-use ultrasound transducers and equipment between patients as well as safe handling and use of ultrasound coupling gel. American Institute of Ultrasound in Medicine. Updated March 27, 2020. https://www.aium.org/officialStatements/57. Accessed 7 Dec 2020 10.1002/jum.16167 36655607

[15] ACEP Policy Statement: Guideline for Ultrasound Transducer Cleaning and Disinfection. Published June 2018. https://www.acep.org/globalassets/new-pdfs/policy-statements/guideline-for-ultrasound-transducer-cleaning-and-disinfection.pdf. Accessed 12 Dec 2020

[16] ACEP guideline on COVID-19: ultrasound machine and transducer cleaning. Published March 2020. https://www.acep.org/globalassets/new-pdfs/policy-statements/guideline-on-covid-19--ultrasound-machine-and-transducer-cleaning.pdf. Accessed 12 Dec 2020 10.1016/j.annemergmed.2020.06.004 PMC7275983 33012406

[17] Basseal J, Westerway S, Juraja M (2017). Guidelines for reprocessing ultrasound transducers. Australas J Ultrasound Med.

[18] Koibuchi H, Kotani K, Taniguchi N (2013). Ultrasound probes as a possible vector of bacterial transmission. Med Ultrason.

[19] Suleyman G, Alangaden G, Bardossy AC (2018). The role of environmental contamination in the transmission of nosocomial pathogens and healthcare-associated infections. Curr Infect Dis Rep.

[20] Hayden MK, Bonten MJ, Blom DW, Lyle EA, van de Vijver DA, Weinstein RA (2006). Reduction in acquisition of vancomycin-resistant enterococcus after enforcement of routine environmental cleaning measures. Clin Infect Dis.

[21] Eckstein BC, Adams DA, Eckstein EC (2007). Reduction of *Clostridium Difficile* and vancomycin-resistant *Enterococcus* contamination of environmental surfaces after an intervention to improve cleaning methods. BMC Infect Dis.

[22] Westerway SC, Basseal JM (2017). The ultrasound unit and infection control—are we on the right track?. Ultrasound.

[23] Westerway SC, Basseal JM, Abramowicz JS (2019). Medical ultrasound disinfection and hygiene practices: WFUMB global survey results. Ultrasound Med Biol.

[24] Hoyer R, Adhikari S, Amini R (2016). Ultrasound transducer disinfection in emergency medicine practice. Antimicrob Resist Infect Control.

[25] Carling PC, Parry MM, Rupp ME (2008). Improving cleaning of the environment surrounding patients in 36 acute care hospitals. Infect Control Hosp Epidemiol.

[26] Goodman ER, Platt R, Bass R, Onderdonk AB, Yokoe DS, Huang SS (2008). Impact of an environmental cleaning intervention on the presence of methicillin-resistant *Staphylococcus aureus* and vancomycin-resistant enterococci on surfaces in intensive care unit rooms. Infect Control Hosp Epidemiol.

[27] Carling PC, Briggs JL, Perkins J, Highlander D (2006). Improved cleaning of patient rooms using a new targeting method. Clin Infect Dis.

[28] Van Kalsbeek D, Enroth K, Benton-Slocum Z, Smith CJ. Cleaning and disinfection of point-of-care ultrasound machines. Published June 2020. https://www.unmc.edu/intmed/residencies-fellowships/cleaning_ultrasound.html. Accessed 24 Jan 2021

[29] Rutala WA, Weber DJ (2008) Guidelines for disinfection and sterilization in healthcare facilities. Center for disease control and prevention

[30] Rupp ME, Fitzgerald T, Sholtz L, Lyden E, Carling P (2014). Maintain the gain: program to sustain performance improvement in environmental cleaning. Infect Control Hosp Epidemiol.

[31] Carling P (2013). Methods for assessing the adequacy of practice and improving room disinfection. Am J Infect Control.

[32] Hassan M, Tuckman HP, Patrick RH, Kountz DS, Kohn JL (2010). Cost of hospital-acquired infection. Hosp Top.

[33] Westphal GA, Pereira AB, Fachin SM (2019). Characteristics and outcomes of patients with community-acquired and hospital-acquired sepsis. Rev Bras Ter Intensiva.

[34] Douglas County Health Department COVID-19 Dashboards (2020) Douglas county health department. https://www.douglascountyhealth.com/infectious-disease/diseases-and-conditions/2019-novel-coronavirus-2019-ncov. Accessed 7 Dec 2020

[35] Grol R, Grimshaw J (2003). From best evidence to best practice: effective implementation of change in patients’ care. Lancet.

