# Supplementary material for: Improving hospital-based point-of-care ultrasound cleaning practices using targeted interventions: a pre–post study
Source: Ultrasound J. 2021 Oct 18;13:43. doi: 10.1186/s13089-021-00244-4 (PMC8522855; doi:10.1186/s13089-021-00244-4)
Supplement: Supplementary file 1 — Additional file 1. Point-of-care ultrasound cleaning survey. [file 13089_2021_244_MOESM1_ESM.docx]

**Additional file 1. Point-of-Care Ultrasound Cleaning Survey**

1. Sex
   1. Male
   2. Female
   3. Intersex
   4. Prefer not to say
2. Level of Training
   1. Medical Student
   2. Resident
   3. Fellow Faculty
   4. Other ______
3. If you are faculty or fellow, what is your primary area of clinical care? (Residents can skip)
   1. General IM – Hospital medicine
   2. General IM – Ambulatory
   3. General IM – Ambulatory and Hospital Medicine
   4. Critical Care Medicine/Pulmonology
   5. Rheumatology
   6. Other ______
4. When on clinical service, how frequently do you use point-of-care ultrasound?
   1. Daily
   2. Weekly
   3. Monthly
   4. Less than once per month
   5. Never
5. When did you first receive formal training in point-of-care ultrasound?
   1. I have never received formal training
   2. As a medical student
   3. As a resident
   4. As a fellow
   5. As a faculty member
   6. Other ______
6. When did you first receive formal training in point-of-care ultrasound CLEANING?
   1. I have never received formal training on point-of-care ultrasound cleaning
   2. As a medical student
   3. As a resident
   4. As a fellow
   5. As a faculty member
   6. Other ______
7. Are you familiar with best practice guidelines for point-of-care ultrasound cleaning?
   1. Yes
   2. No
8. Do you know where to find Nebraska Medicine’s policies covering point-of-care ultrasound cleaning?
   1. Yes
   2. No
9. What is your level of agreement/disagreement with the following statement: My point-of-care ultrasound cleaning practices follow best practice guidelines.
   1. Strongly agree
   2. Agree
   3. Don’t know
   4. Disagree
   5. Strongly Disagree
10. What is your level of agreement/disagreement with the following statement: Following point-of-care ultrasound cleaning guidelines improves patient safety and health.
    1. Strongly agree
    2. Agree
    3. Don’t know
    4. Disagree
    5. Strongly Disagree
11. What is/are the recommended low-level disinfectant(s) for ultrasound machines at UNMC? Check all those that apply.
    1. 70% isopropyl alcohol
    2. 60% ethyl alcohol
    3. Sani-cloth AF3 wipes (Gray top tub)
    4. Sani-cloth bleach wipes (Orange top tub)
    5. Pressurized steam
    6. Don’t know
12. Which of the following should be performed using a sterile probe cover? Choose all that apply.
    1. Cardiac exam
    2. Central venous catheter placement
    3. Marking for a paracentesis
    4. Diagnostic lung ultrasound
    5. Arterial line placement
    6. Don’t know
13. Which of the following best describes the standard 2-step process for ultrasound equipment cleaning following an exam on intact skin?
    1. Disinfection followed by storage
    2. Disinfection followed by removal of gross debris
    3. Turn the machine off followed by disinfection
    4. Removal of gross debris followed by disinfection
    5. Low-level disinfection, followed by high-level disinfection process
    6. Don’t know
14. Which parts of the ultrasound equipment should be disinfected following an exam on intact skin? Choose all those that apply
    1. Probe(s) used during the exam
    2. Probes(s) not used during the exam
    3. Probe cord(s) used during the exam
    4. Probe cord(s) not used during the exam
    5. Monitor front
    6. Monitor back
    7. Cart push handle
    8. Console surface (eg touch pad, track ball, keyboard)
    9. Wheel casing
    10. Power cord
    11. Don’t know
15. How frequently do you encounter the following barriers when cleaning ultrasound machines? *(Each of the following had 6 options, allowing participants to choose 1: Never, Rarely, Sometimes, Often, Always, N/A)*
    1. Limited access to cleaning supplies
    2. Time constraints
    3. Lack of knowledge in machine cleaning procedures
    4. Forgetfulness
    5. Lack of motivation to integrate cleaning procedures into my routine
16. If you encounter barriers other than those listed above please list them here.
    1. ______
